# *Senecio changii* (Asteraceae: Senecioneae), a New Species from Sichuan, China

**DOI:** 10.1371/journal.pone.0151423

**Published:** 2016-04-06

**Authors:** Chen Ren, Tian-Jing Tong, Yu Hong, Qin-Er Yang

**Affiliations:** 1 Key Laboratory of Plant Resources Conservation and Sustainable Utilization, South China Botanical Garden, Chinese Academy of Sciences, Guangzhou 510650, People’s Republic of China; 2 Key Laboratory of Plant Resources Conservation and Utilization of Hunan Province, Jishou University, Jishou 416000, People’s Republic of China; 3 University of Chinese Academy of Sciences, Beijing 100049, People’s Republic of China; Institute of Botany, CHINA

## Abstract

*Senecio changii* (Asteraceae: Senecioneae), a new species from Muli, Sichuan, southwestern China, is described. It is distinguished in Chinese *Senecio* s.s. by having lyrate-pinnatisect to pinnatisect leaves and a single terminal large discoid capitulum which is somewhat nodding. Evidence from floral micromorphology, karyology and molecular phylogenetic analyses based on the nuclear ITS/ETS sequence data all support its membership within *Senecio* s.s.

## Introduction

*Senecio* L. (Asteraceae: Senecioneae), as recently delimitated [1, 2], consists of ca. 1,000 species with an almost cosmopolitan distribution. The genus is not particularly richly represented in China. In the *Flora of China* 20–21 published in 2011, 65 species were recorded in the genus [3]. These include ten species that have been transferred to *Jacobaea* Mill. [4–6], one to *Madaractis* DC. [1, 2], and three (all within *S*. sect. *Flexicaules* C. Jeffrey & Y.L. Chen) that represent an independent genus of their own but not as yet formally named [1]. Recently, *S*. *daochengensis* Y.L. Chen was synonymized with *S*. *atrofuscus* Grierson [7]. Fifty species in total, therefore, are currently recognized in *Senecio* s.s. from China. They are mostly distributed in the Hengduan Mountains region in southwestern China, one of the biodiversity hotspots in the world [8, 9].

**Fig 1:**
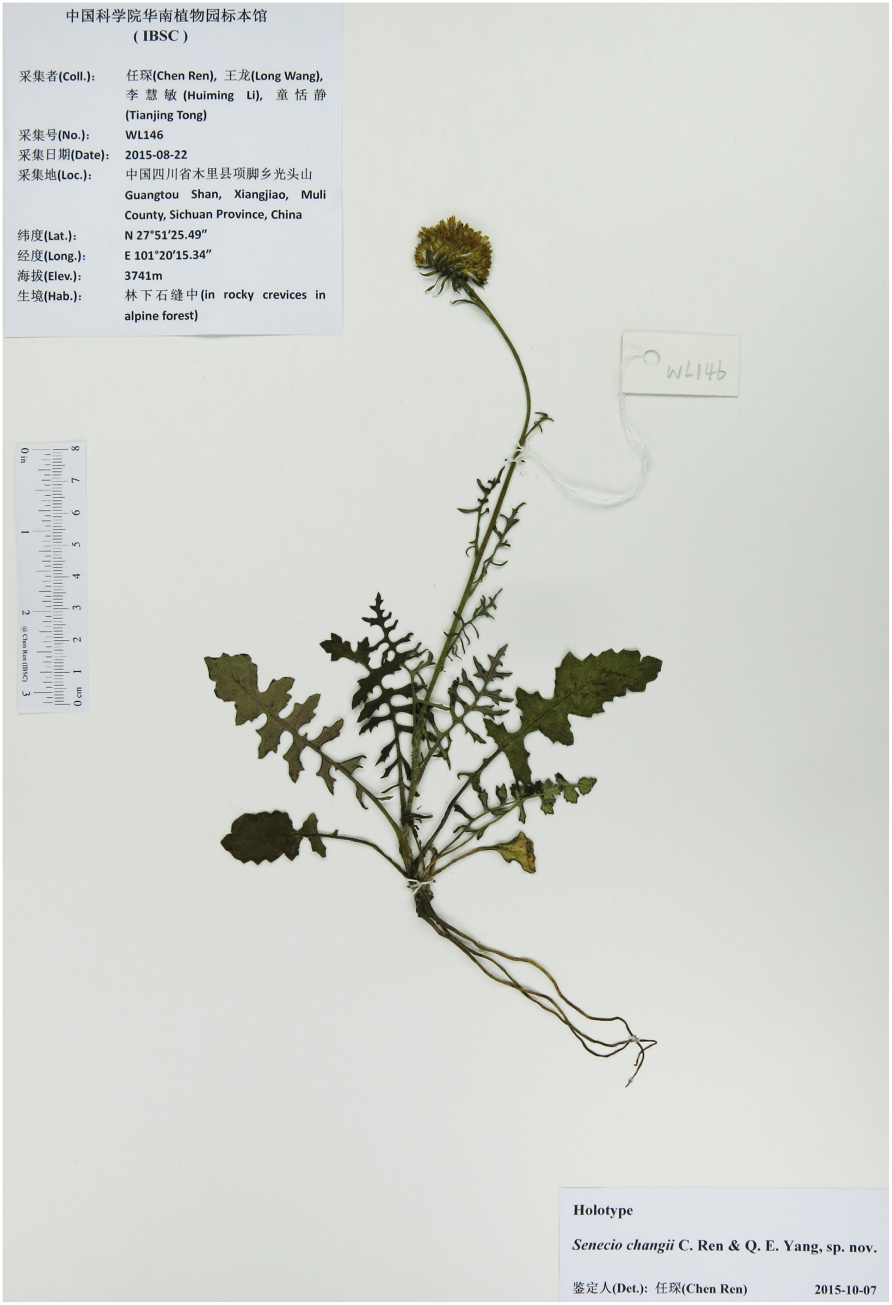
Holotype sheet of *Senecio changii*.

**Fig 2:**
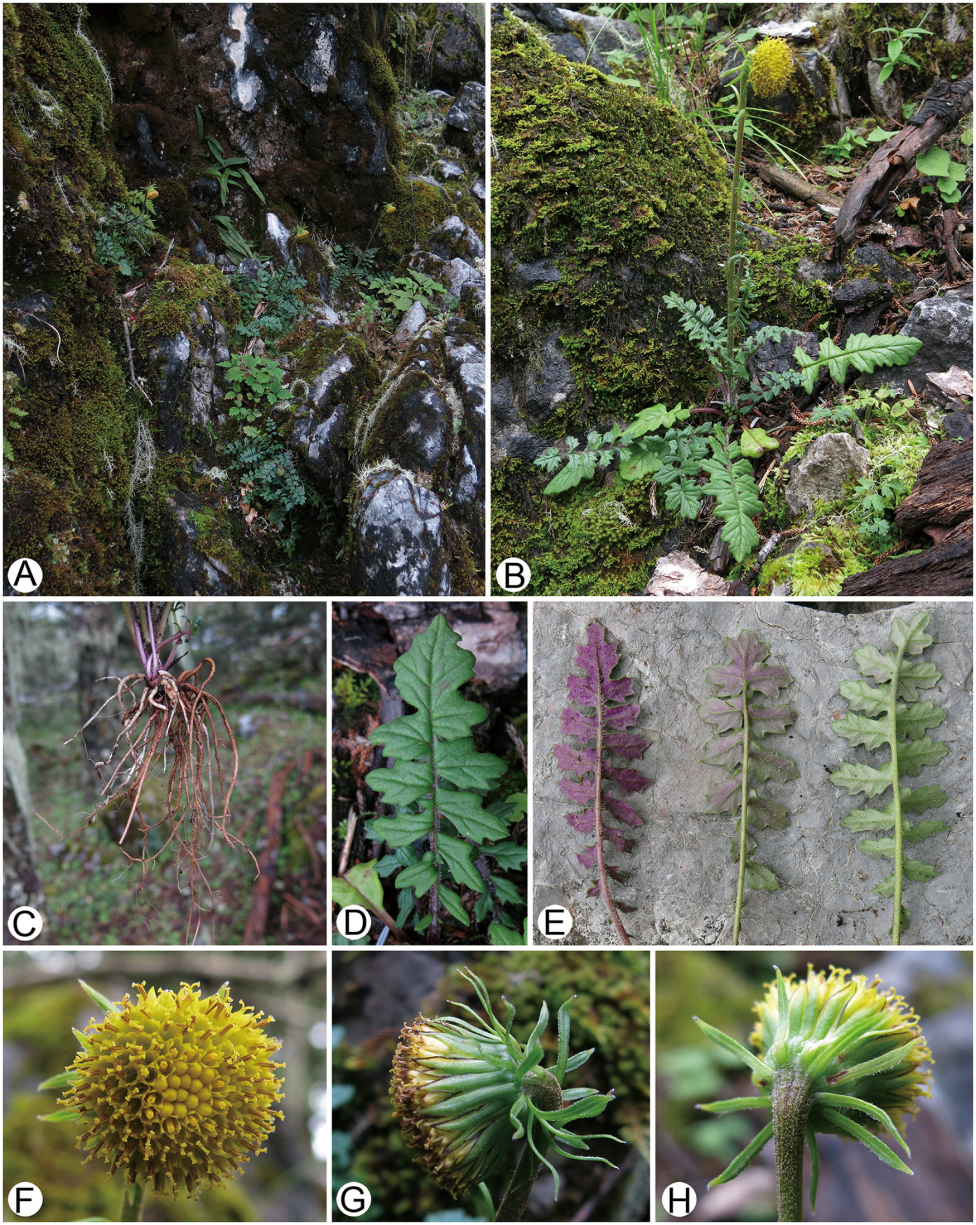
*Senecio changii* in the wild (Guangtou Shan, Muli, Sichuan, China). (A) Habitat; (B) Habit; (C) Roots; (D) Leaf (adaxial surface); (E) Leaves (abaxial surface) (F) Capitulum (top view); (G) Capitulum (lateral view); (H) Capitulum (back view).

During a botanical expedition to Sichuan Province in southwestern China in 2015, we discovered an unusual population of *Senecio* s.s. in Muli County, an area situated in the southern part of the Hengduan Mountains region. The plants are most readily distinguishable from all the other known Chinese species in *Senecio* s.s. by having lyrate-pinnatisect or pinnatisect leaves and a single terminal large discoid capitulum which is somewhat nodding (Figs 1 and 2). We determined that the population represents a hitherto undescribed species, which we name as *S*. *changii* and describe below. Its membership within *Senecio* s.s. is strongly supported by evidence from floral micromorphology, karyology and molecular phylogenetic analyses based on the nuclear rDNA internal and external transcribed spacer (ITS and ETS) sequences.

## Materials and Methods

### Ethics statements

The new species reported in this study was collected from Guangtou Shan in Muli County, Sichuan Province, China. The collection locality is situated in a state forest farm superintended by the Forestry Bureau of Sichuan Province. The Forestry Bureau permitted our field studies in the farm, and our field studies did not involve endangered or protected species.

### Morphology

The morphological description of the new species was based on the examination of fresh and pressed specimens. For morphological comparison with its more or less similar species, including *Senecio chungtienensis* C. Jeffrey & Y.L. Chen, *S*. *megalanthus* Y.L. Chen, and *S*. *pteridophyllus* Franch., relevant specimens deposited in the major Chinese herbaria, including CDBI, IBSC, KUN, PE, SM, SZ, and WUK, were examined.

### Floral micromorphology

Thirty florets from ten individuals of *Senecio changii* were examined. Dry mature florets were first boiled in distilled water for 20 min. For observation of style-arm apices and stigmatic areas on the inner surface of the style branch, styles were segregated from the florets and mounted on a slide. For observation of the filament collar and anther endothecial cell wall thickenings, softened florets were immersed in 5% NaOH at 45°C for 1 h, then the anthers were removed from the florets and mounted on a slide with 50% glycerol. All the observations were made under a Nikon microscope (ECLIPSE E600), and photographs were made with a Nikon digital camera (DXM1200F).

Terminology for the description of the configuration of stigmatic areas on the inner surface of the style branch followed Wetter [10], and that for the description of the filament collar and of the endothecial cell wall thickenings followed Nordenstam [11].

### Karyology

Five plant individuals of *Senecio changii* were examined. Root tips were pretreated in a 1:1 mixture of 0.1% colchicine and 0.002 M 8-hydroxyquinoline for 2.5 h, then fixed in Carnoy I (glacial acetic acid: absolute ethanol = 1:3) at room temperature for 1 h, and then macerated in a 1:1 mixture of 45% acetic acid and 1 M HCl at 37°C for 45 min, and stained and squashed in Carbol fuchsin. Photographs of chromosomes were made with a Nikon microscope (ECLIPSE E600) with a Nikon digital camera (DXM1200F).

The karyotype formula was based on measurements of chromosomes of five cells. The acronyms used to describe the karyotypes followed Levan et al. [12]: m = median centromeric chromosome with arm ratio of 1.0–1.7, and sm = submedian centromeric chromosome with arm ratio of 1.7–3.0.

### Molecular systematics

#### Taxon sampling

A total of 67 accessions were sampled, including three accessions of *S*. *changii*, 29 accessions representing 29 species within *Senecio* s.s., and 35 accessions in 35 genera representing other main clades of Senecioneae revealed by Pelser et al. [1, 13]. *Abrotanella emarginata* (Gaudich.) Cass. was selected as a root based on Pelser et al. [1, 13]. GenBank accession numbers and voucher information for the materials used in this study are provided in S1 Table.

#### DNA extraction, amplification and sequencing

Total genomic DNA of *Senecio changii* was extracted from silica gel-dried leaves collected in the field using a CTAB protocol [14]. ITS and ETS sequence data were employed to infer phylogeny in this study. They are amplified and sequenced using primer pairs ITS4 (TCCTCCGCTTATTGATATGC)/ITS5 (GCAAGTAAAAGTCGTAACAAGG) [15] and AST-1 (CGTAAAGGTGCATGAGTGGTGT) [16]/18S-ETS (ACTTACACATGCATGGCTTAATCT) [17], respectively. Polymerase chain reactions (PCRs) were performed in a total volume of 25 μl containing 5 μl 5× PrimeSTAR buffer (Mg^2+^ plus) (Takara, Dalian, Liaoning, China), 0.2 mM each dNTP, 0.3 μM each primer, 50 ng template DNA, and 0.6 U PrimeSTAR HS DNA Polymerase (Takara, Dalian, Liaoning, China). PCR conditions followed the suggestions of the user manual of PrimeSTAR HS DNA Polymerase: a pretreatment at 98°C for 3 min, 30 cycles of DNA denaturation at 98°C for 10 s, primer annealing at 55°C for 15 s, and DNA extension at 72°C for 30 s, followed by a single final extension at 72°C for 6 min. PCR products were visualized via agarose gel electrophoresis. Successful amplifications were purified, and sequenced on an Applied Biosystems 3730xl DNA Analyzer at Invitrogen (Life Technologies, Guangzhou, Guangdong, China). The resulting contigs were assembled and edited using Sequencher v.4.1.4 (Gene Codes Corporation, Ann Arbor, Michigan, U.S.A.). Six sequences of *Senecio changii* were deposited at GenBank (S1 Table).

#### Phylogenetic analyses

Sequences were aligned using Clustal X v.2.1 [18] with the default settings, and followed by manual adjustment in BioEdit v.7.0.5.3 [19] when necessary. The concatenated alignment of ITS and ETS could be found in S1 File (ITS: 1–950; ETS: 951–1058). Gaps introduced for alignment were treated as missing.

Before combining ITS and ETS for phylogenetic analyses, we performed incongruence length difference (ILD) test [20, 21] to test the congruence between them. The ILD test was implemented in PAUP* v.4.0b10 [22] with 1,000 replicates, each using a heuristic search algorithm with 100 random-addition-sequence replicates, tree bisection-reconnection (TBR) branch-swapping, and saving multiple trees. *P*-values below 0.05 indicated significant incongruence [20, 21].

Maximum parsimony (MP), maximum likelihood (ML) and Bayesian inference (BI) methods were employed to infer the phylogeny. MP analysis was performed using PAUP* v.4.0b10 [22] with the following settings: heuristic tree search, 10,000 random-addition-sequence replicates, TBR branch-swapping, saving multiple trees. Node support was estimated with 1,000 bootstrap (MPBS) replicates [23], each using a heuristic search algorithm with 100 random-addition-sequence replicates, TBR branch-swapping, and saving multiple trees. For ML and BI analyses, ITS and ETS were assigned separate partitions. ML analysis was implemented in RAxML v.8.1.20 [24] with GTR+GAMMA model assigned for both partitions and with 1,000 bootstrap (MLBS) replicates using a fast bootstrapping algorithm [25]. MrBayes v.3.2.5 [26] was employed to implement BI. We sampled across the GTR model space (nst = mixed) [27] with a gamma-shaped distribution of rates across sites. All parameters were unlinked and variable rates were allowed. Two parallel analyses each with four chains were run for three million generations. Trees were sampled every 1,000 generations. Average standard deviation of split frequencies below 0.01, and effective sample sizes (ESS) of all parameters over 200 evaluated in Tracer v.1.6 [28], were used as indicators of convergence and adequate sampling. The first 25% sampled trees were discarded as burn-in, and the remaining trees were used to estimate the posterior probabilities (PP). Bootstrap percentage (MPBS and MLBS) values ≥ 70 [29] and PP values ≥ 0.95 were regarded as strong support.

### Nomenclature

The electronic version of this article in Portable Document Format (PDF) in a work with an ISSN or ISBN will represent a published work according to the International Code of Nomenclature for algae, fungi, and plants, and hence the new name contained in the electronic publication of a PLOS article is effectively published under that Code from the electronic edition alone, so there is no longer any need to provide printed copies.

In addition, the new name contained in this work has been submitted to IPNI, from where it will be made available to the Global Names Index. The IPNI LSIDs can be resolved and the associated information viewed through any standard web browser by appending the LSID contained in this publication to the prefix http://ipni.org/. The online version of this work is archived and available from the following digital repositories: PubMed Central and LOCKSS.

## Results

### Floral Micromorphology

**Fig 3:**
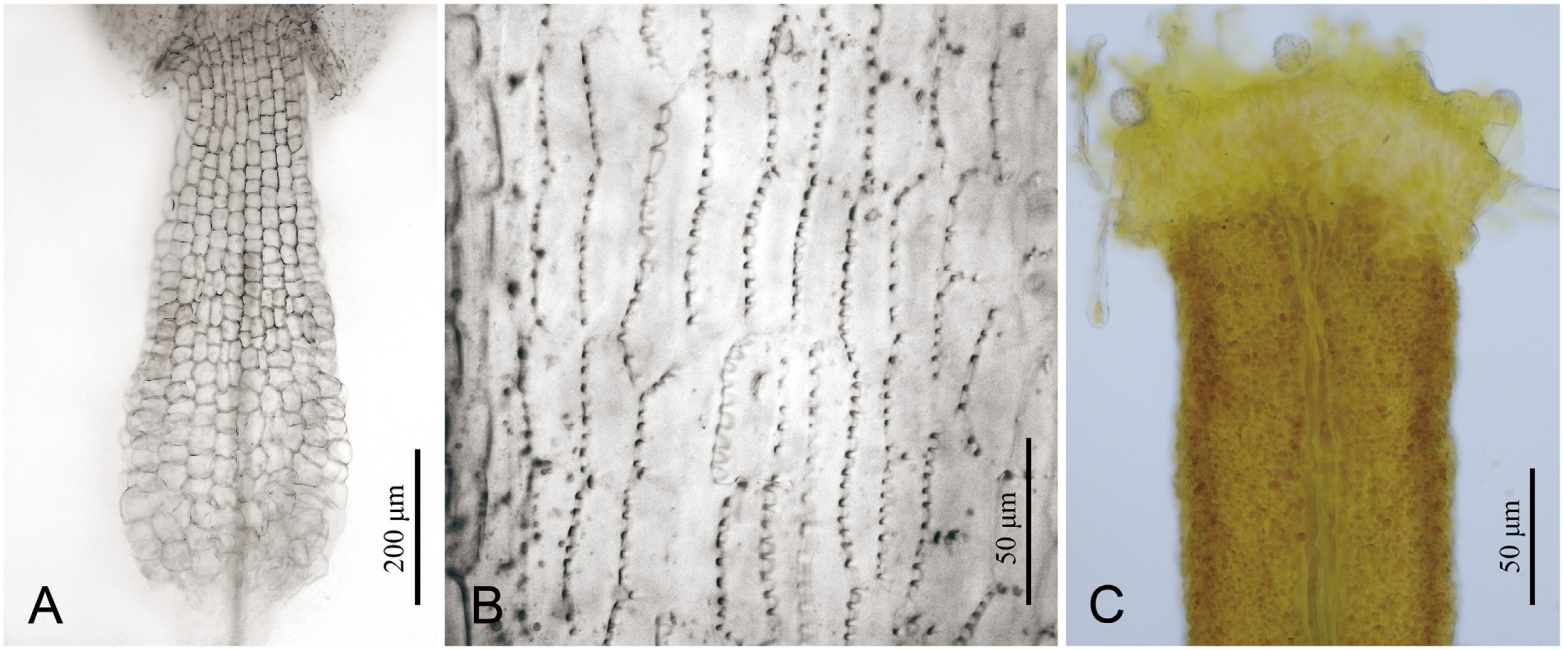
Floral micromorphology in *Senecio changii*. (A) Filament collar, balusterform; (B) Anther endothecial cell wall thickenings, radial; (C) Style arm, showing the truncate apex and discrete stigmatic areas.

The filament collar of *Senecio changii*, which was dilated towards the base with the basal cells somewhat enlarged, was balusterform (Fig 3A). The anther endothecial cell wall thickenings were radial, with the thickenings or ribs nearly evenly distributed in the inner anticlinal walls (Fig 3B). The style-arm apices were truncate and the stigmatic areas on the inner surface of the style branch were discrete (Fig 3C).

### Karyology

**Fig 4:**
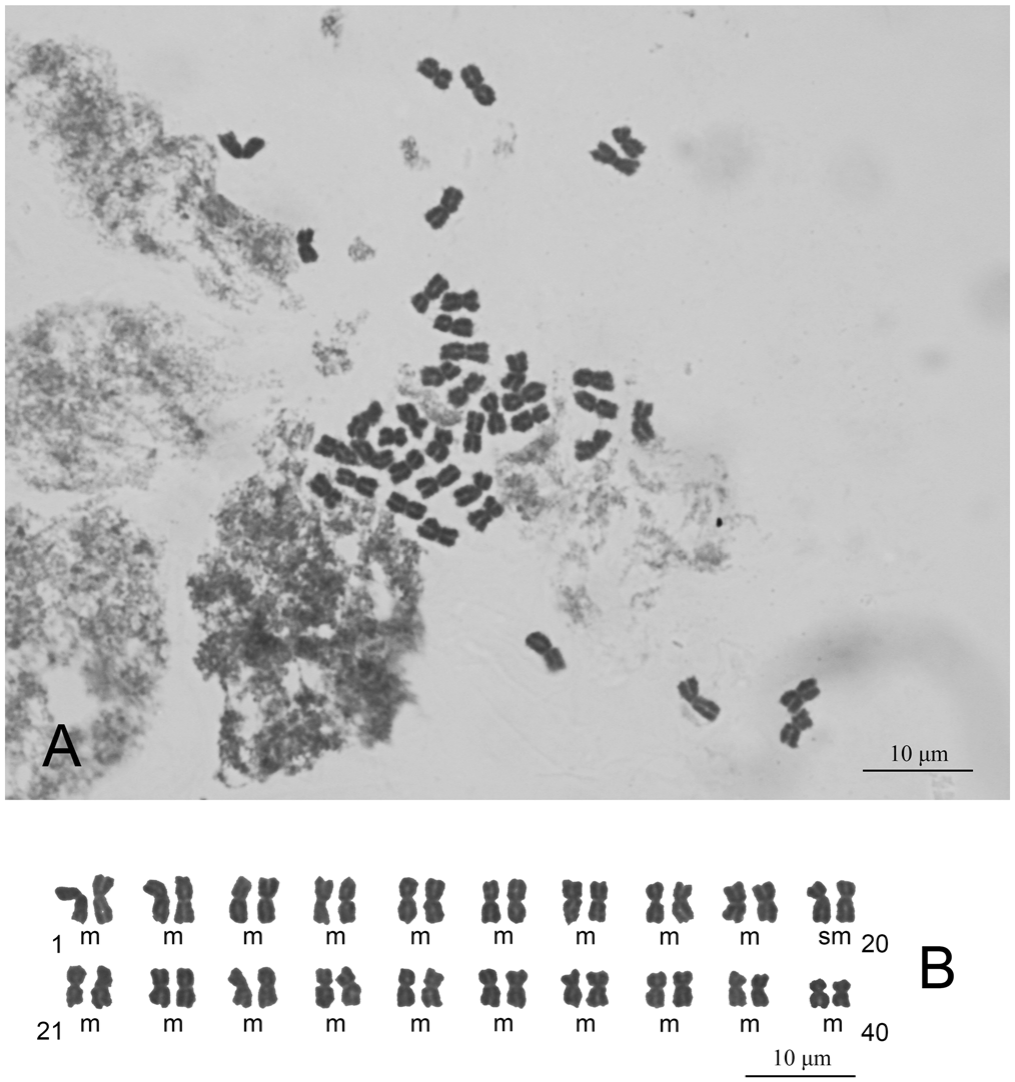
Mitotic metaphase chromosomes (A; 2*n* = 40) and karyotype (B; 2*n* = 38m + 2sm) of *Senecio changii*.

The chromosome number of *Senecio changii* was 2*n* = 40 (Fig 4A). The chromosomes were medium-sized, ranging from 4.6 to 2.6 μm in length. They showed a steady gradation in length from the longest to the shortest, with no evidence of bimodality. The karyotype was formulated as 2*n* = 38m + 2sm (Fig 4B).

### Molecular Systematics

The *p*-value of the ILD test between ITS and ETS data was 0.127, indicating that there was no significant incongruence between these two regions. We thus combined them for phylogenetic analyses.

**Fig 5:**
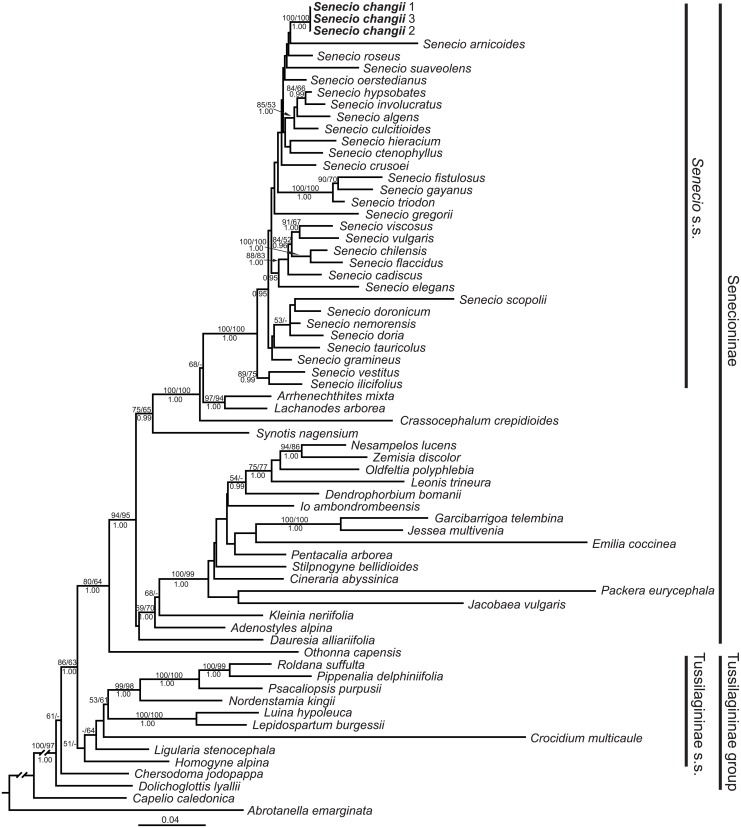
Phylogeny of tribe Senecioneae based on concatenated ITS and ETS data by using maximum likelihood analysis. Bootstrap values (≥ 50%; maximum likelihood/maximum parsimony) are indicated above branches, and posterior probabilities (≥ 0.95) below branches. Dashes (-) indicate bootstrap values < 50%.

MP (S1 Fig), ML (Fig 5) and BI (S2 Fig) analyses all produced congruent topologies, except for the position of *Crocidium multicaule* Hook. This species was suggested as a member of subtribe Tussilagininae s.s. by both ML and BI analyses, although not receiving strong support, while in MP tree, it was sister to *Othonna capensis* L.H. Bailey (MPBS = 79).

As shown in Fig 5, after *Abrotanella emarginata*, *Capelio caledonica* B. Nord. and three lineages of the subtribe Tussilagininae grade successively diverged. Subtribe Senecioninae was resolved as a well-supported clade (MLBP/MPBP/PP = 94/95/1.00), with *Othonna capensis* as a sister (MLBP/MPBP/PP = 80/64/1.00). Within Senecioninae, the monophyly of *Senecio* s.s. was strongly supported (MLBP/MPBP/PP = 100/100/1.00), although phylogenetic relationships within the subtribe were poorly resolved. Significantly, the three accessions of *Senecio changii* constituted a strongly-supported clade (MLBP/MPBP/PP = 100/100/1.00) deeply nested within *Senecio* s.s.

### Taxonomic treatment

***Senecio changii*** C. Ren & Q.E. Yang, **sp. nov.** [urn:lsid:ipni.org:names: 77153507–1] (Figs 1–3) Type: China. Sichuan: Muli, Xiangjiao, Guangtou Shan, 27°51′25.49″ N, 101°20′15.34″ E, 3741 m, rocky crevices in alpine forest, 22 Aug 2015 (fl), *C*. *Ren et al*. *WL146* (holotype and isotypes, IBSC!).

#### Diagnosis

*Senecio changii* is distinguished in Chinese *Senecio* s.s. by having lyrate-pinnatisect to pinnatisect leaves and a single large terminal discoid capitulum which is somewhat nodding.

#### Description

Herbs, perennial, rhizomatous. Stem solitary, erect, scapiform, 10–20 cm tall, crisped-pubescent, especially densely so near base. Basal leaves many, present at anthesis; petiole 2–6 cm long, crisped-pubescent, base dilated but not auriculate; blade oblanceolate-oblong or narrowly oblanceolate-oblong, 4–10 × 1.2–3 cm, herbaceous, lyrate-pinnate to pinnate with irregularly sparsely dentate lobes, adaxially green, abaxially whitish green to purple, crisped-pubescent on both surfaces, especially so at margins and on veins. Stem leaves several, similar to basal leaves and gradually smaller upwards. Capitula solitary, discoid, slightly nodding; scape 7–11 cm tall, crisped-pubescent, with 2–4 linear bracts in upper part. Involucres campanulate, 10–14 × 15–20 mm, conspicuously calyculate; bracts of calyculus 12–18, linear, 10–14 × 2–3 mm, herbaceous; phyllaries 20–26, linear, 10–14 × 2–3 mm, herbaceous, crisped-pubescent, apically purplish, puberulent. Disk florets numerous; corolla yellow, 7–8 mm long, with ca. 2.5 mm tube and funnelform limb; lobes oblong-lanceolate, ca. 1 mm long, apically acute, papillose; anthers ca. 1.5 mm long, basally obtuse-auriculate, appendages ovate-lanceolate; style branches ca. 0.5 mm long, apically truncate; stigmatic areas separate. Achenes (immature) cylindric. Pappus white, ca. 4 mm long.

#### Etymology

The epithet “*changii*” is named in honor of the late Professor Chao-chien Chang (1900–1972), who was one of the Chinese pioneers in taxonomic studies of the Asteraceae from China.

#### Distribution and Habitat

**Fig 6:**
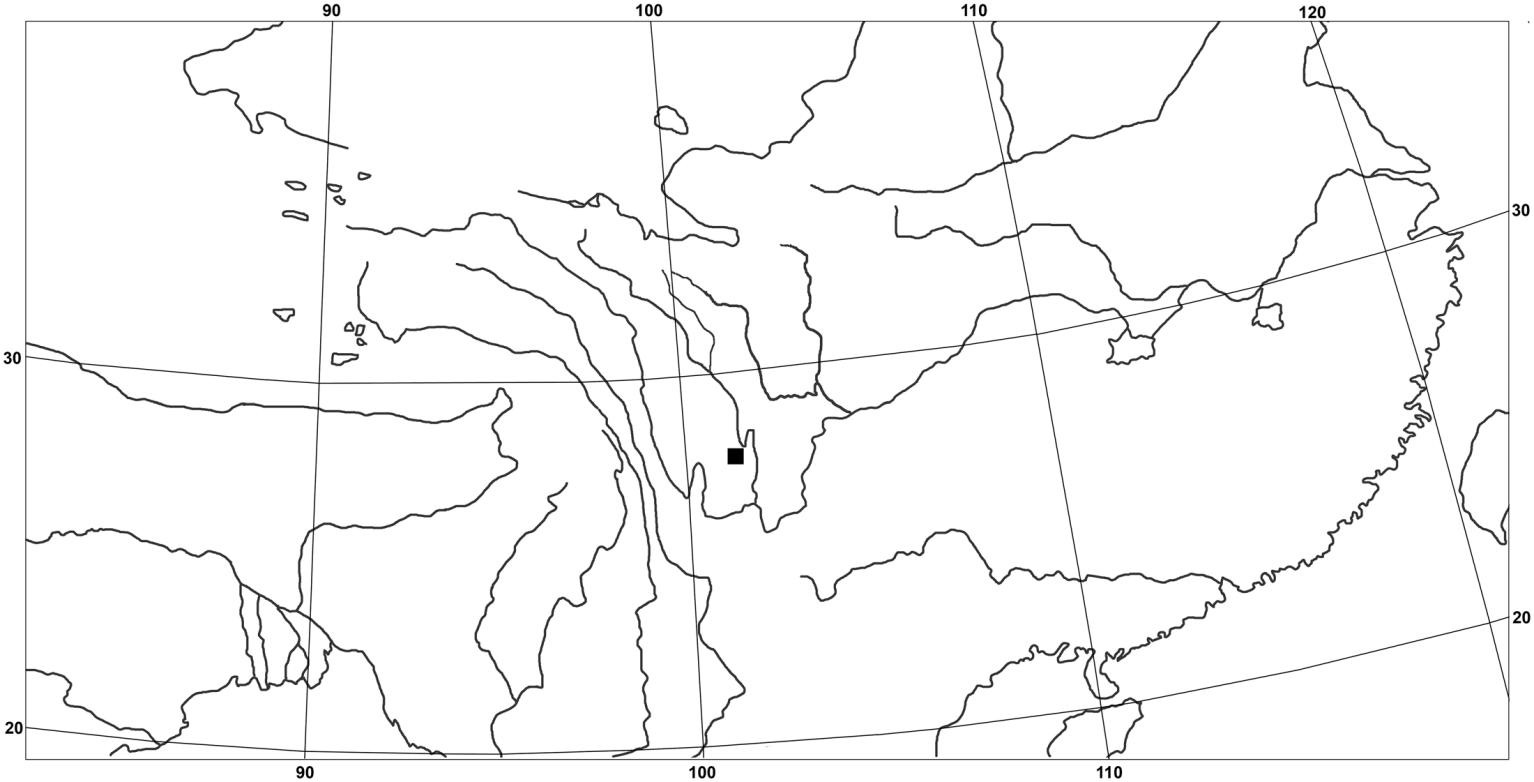
Distribution of *Senecio changii*.

*Senecio changii* is currently known only from its type locality, i.e., Guangtou Shan, Muli, Sichuan, China (Fig 6). It is a chasmophyte growing in rocky crevices in alpine forest at an altitude of ca. 3700 m a.s.l.

#### Phenology

Found in flower in late August.

#### Conservation Status

According to the IUCN red list categories and criteria, Version 3.1 [30], *Senecio changii* should be categorized as a Critically Endangered (CR) species. This species seems to be quite rare. It had never been collected before, and we discovered only a small population of some 150 mature individuals despite our great efforts to find more populations. In addition, *S*. *changii* seems to prefer highly specialized habitats like rocky crevices, and is thus prone to threats from human activities such as grazing and mining. Fortunately, the type locality is not easily accessible and sparsely populated. The population seems to be reproductively healthy, with 1/3 of the individuals are in blossom. The presence of many seedlings in the population indicates that the species should be sexually reproductive and its seeds can germinate normally.

### Key to Similar Species

1a Capitula solitary, 10–14 × 15–20 mm.
  2a Leaves lyrate-pinnatisect to pinnatisect; capitula discoid………..*Senecio changii*
  2b Leaves subentire or undulate-dentate; capitula radiate…………….*S*. *megalanthus*
1b Capitula numerous in a compound corymb, 3–6 × 2–4 mm.
  3a Rays 4.5 mm long; pappus 4 mm long…………………………*S*. *pteridophyllus*
  3b Rays 8 mm long; pappus 5.5 mm long……………………….. *S*. *chungtienensis*

## Discussion

The generic affiliation of *Senecio changii* is strongly supported by evidence from gross morphology, floral micromorphology, karyology, and ITS/ETS sequence data. The somewhat nodding capitulum of *S*. *changii* is reminiscent of members within *Cremanthodium* Benth. within subtribe Tussilagininae. However, the calyculate involucre with numerous bracts (Fig 2F–2H), the balusterform filament collar (Fig 3A), the radial anther endothecial cell wall thickenings (Fig 3B), the truncate style-arm apices (Fig 3C), the discrete stigmatic areas (Fig 3C), and the chromosome number of 2*n* = 40 (Fig 3) all indicate that *S*. *changii* is a member within subtribe Senecioninae as defined by Nordenstam [31]. In subtribe Tussilagininae, the involucre is usually ecalyculate, the filament collar is cylindric, the endothecial cell wall thickenings are polarized, the style-arm apices are obtuse, the stigmatic areas are continuous, and the base chromosome number is *x* = 30 or a derivative thereof [31]. In our molecular phylogenetic analyses based on ITS/ETS sequence data, *Senecio changii* is revealed to be deeply nested within *Senecio* s.s. (Fig 5).

*Senecio changii* is morphologically a very distinctive species in Chinese *Senecio* s.s. in having lyrate-pinnatisect to pinnatisect leaves and a single terminal large discoid capitulum which is somewhat nodding. Although *S*. *megalanthus*, a species occurring in Xiangcheng and Kangding in western Sichuan and belonging in *S*. sect. *Crociseris* (Reichenb.) Hall. & Wohlf. ser. *Monticolarum* C. Jeffrey & Y.L. Chen, also has a single terminal large capitulum, but from *S*. *changii* it immediately differs by the leaves subentire or undulate-dentate and the capitulum radiate. From a morphological perspective *S*. *changii* may be referred to *S*. sect. *Crociseris* ser. *Malacophylli* C. Jeffrey & Y.L. Chen because of its scapiform habit and lyrate-pinnatisect to pinnatisect leaves. In leaf shape, particularly, *S*. *changii* is similar to *S*. *pteridophyllus* and *S*. *chungtienensis* within the series. However, both *S*. *pteridophyllus* and *S*. *chungtienensis* occur in northwestern Yunnan, China, and are most easily distinguishable from *S*. *changii* by having a compound corymb composed of numerous small radiate capitula which are erect.

## Supporting Information

S1 Fig Phylogeny of tribe Senecioneae based on concatenated ITS and ETS data by using maximum parsimony analysis. Bootstrap values (≥ 50%) are indicated above branches.
(EPS)

S2 Fig Phylogeny of tribe Senecioneae based on concatenated ITS and ETS data by using Bayesian analysis. Posterior probabilities (≥ 0.95) are indicated above branches.
(EPS)

S1 File The concatenated ITS and ETS dataset. ITS region: 1–950; ETS region: 951–1058.
(FAS)

S1 Table GenBank accession numbers and voucher information for the materials used in this study. (XLSX)

## References

[1] Pelser PB, Nordenstam B, Kadereit JW, Watson LE. An ITS phylogeny of tribe Senecioneae (Asteraceae) and a new delimitation of *Senecio* L. Taxon. 2007; 56: 1077–1104.

[2] Nordenstam B, Pelser PB, Kadereit JW, Watson LE. Tribe Senecioneae In Funk VA, Susanna A, Stuessy TF, Bayer RJ, editors. Systematics, evolution, and biogeography of the Compositae. Vienna: International Association for Plant Taxonomy; 2009 pp. 503–525.

[3] Chen YL, Liu SW, Lu Y, Yang QE, Nordenstam B, Illarionova ID, et al Tribe Senecioneae In: Wu ZY, Raven PH, editors. Flora of China, Vol. 20–21. Beijing: Science Press and St. Louis: Missouri Botanical Garden Press; 2011 pp. 371–544.

[4] Pelser PB, Gravendeel B, Meijden RVD. Tackling speciose genera: species composition and phylogenetic position of *Senecio sect*. *Jacobaea* (Asteraceae) based on plastid and nrDNA sequences. Am J Bot. 2002; 89: 929–939. 10.3732/ajb.89.6.929 21665692

[5] Pelser PB, Veldkamp JF, Meijden RVD. New combinations in *Jacobaea* Mill. (Compositae-Senecioneae). Comp Newslett. 2006; 44: 1–11.

[6] Nordenstam B. Additions to the genus Jacobaea Mill. (Compositae-Senecioneae). Comp Newslett. 2006; 44: 12–13.

[7] Tang M, Yang QE. The identity of *Senecio daochengensis* (Asteraceae-Senecioneae). J Trop Subtrop Bot. 2013; 21: 220–224.

[8] Myers N, Mittermeier RA, Mittermeier CG, Fonseca GABD, Kent J. Biodiversity hotspots for conservation priorities. Nature. 2000; 403: 853–858. 10706275 10.1038/35002501

[9] Mittermeier RA, Robles Gil P, Hoffman M, Pilgrim J, Brooks T, Mittermeier CG, et al Hotspots revisited. Mexico City: CEMEX; 2004.

[10] Wetter M. Micromorphological characters and generic delimitation of some New World Senecioneae (Asteraceae). Brittonia. 1983; 35: 1–22.

[11] Nordenstam B. Taxonomic studies in the tribe Senecioneae (Compositae). Opera Bot. 1978; 44: 1–83.

[12] Levan A, Fredga K, Sandberg AA. Nomenclature for centromeric position on chromosomes. Hereditas. 1964; 52: 201–220.

[13] Pelser PB, Kennedy AH, Tepe EJ, Shidler JB, Nordenstam B, Kadereit JW, et al Patterns and causes of incongruence between plastid and nuclear Senecioneae (Asteraceae) phylogenies. Am J Bot. 2010; 97: 856–873. 10.3732/ajb.0900287 21622451

[14] Doyle JJ, Doyle JL. A rapid DNA isolation procedure for small quantities of fresh leaf tissue. Phytochem Bull. 1987; 19: 11–15.

[15] White TJ, Bruns T, Lee S, Taylor J. Amplification and direct sequencing of fungal ribosomal RNA genes for phylogenetics In: Innis MA, Gelfand DH, Sninsky JJ, White TJ, editors. PCR protocols: a guide to methods and applications. San Diego: Academic Press; 1990 pp. 315–322.

[16] Markos S, Baldwin BG. Higher-level relationships and major lineages of *Lessingia* (Compositae, Astereae) based on nuclear rDNA internal and external transcribed spacer (ITS and ETS) sequences. Syst Bot. 2001; 26: 168–183.

[17] Baldwin BG, Markos S. Phylogenetic utility of the external transcribed spacer (ETS) of 18S-26S rDNA: congruence of ETS and ITS trees of *Calycadenia* (Compositae). Mol Phylogenet Evol. 1998; 10: 449–463. 10051397 10.1006/mpev.1998.0545

[18] Larkin MA, Blackshields G, Brown NP, Chenna R, McGettigan PA, McWilliam H, et al Clustal W and Clustal X version 2.0. Bioinformatics. 2007; 23: 2947–2948. 17846036 10.1093/bioinformatics/btm404

[19] Hall TA. BioEdit: a user friendly biological sequence alignment editor and analysis program for Windows 95/98/NT. Nucleic Acids Symp Ser. 1999; 45: 95–98.

[20] Farris JS, Källersjö M, Kluge AG, Bult C. Constructing a significance test for incongruence. Syst Biol. 1995; 44: 570–572.

[21] Farris JS, Källersjö M, Kluge AG, Bult C. Testing significance of incongruence. Cladistics. 1995; 10: 315–319.

[22] Swofford DL. PAUP*: Phylogenetic analysis using parsimony (*and other methods), version 4.0b10. Sunderland, Massachusetts: Sinauer; 2003.

[23] Felsenstein J. Confidence limits on phylogenies: an approach using the bootstrap. Evolution. 1985; 39: 783–791. 28561359 10.1111/j.1558-5646.1985.tb00420.x

[24] Stamatakis A. RAxML version 8: a tool for phylogenetic analysis and post-analysis of large phylogenies. Bioinformatics. 2014; 30: 1312–1313. 10.1093/bioinformatics/btu033 24451623 PMC3998144

[25] Stamatakis A, Hoover P, Rougemont J. A rapid bootstrap algorithm for the RAxML web servers. Syst Biol. 2008; 57: 758–771. 10.1080/10635150802429642 18853362

[26] Ronquist F, Teslenko M, Mark PVD, Ayres DL, Darling A, Höhna S, et al MrBayes 3.2: efficient Bayesian phylogenetic inference and model choice across a large model space. Syst Biol. 2012; 61: 539–542. 10.1093/sysbio/sys029 22357727 PMC3329765

[27] Huelsenbeck JP, Larget B, Alfaro ME. Bayesian phylogenetic model selection using reversible jump Markov chain Monte Carlo. Mol Biol Evol. 2004; 21: 1123–1133. 15034130 10.1093/molbev/msh123

[28] Rambaut A, Suchard MA, Xie D, Drummond AJ. Tracer v1.6. 2014. Available: http://beast.bio.ed.ac.uk/Tracer.

[29] Hillis DM, Bull JJ. An empirical test of bootstrapping as a method for assessing confidence in phylogenetic analysis. Syst Biol. 1993; 42: 182–192.

[30] IUCN. IUCN red list categories and criteria: version 3.1. Second edition Gland, Switzerland and Cambridge, UK: IUCN; 2012.

[31] Nordenstam B. Senecioneae and Liabeae–systematic review In: Heywood VH, Harborne JB, Turner BL, editors. The Biology and chemistry of the Compositae, Vol. 2. London: Academic Press; 1977 pp. 799–830.

